# Long-term continuous mono-cropping of *Macadamia integrifolia* greatly affects soil physicochemical properties, rhizospheric bacterial diversity, and metabolite contents

**DOI:** 10.3389/fmicb.2022.952092

**Published:** 2022-10-06

**Authors:** Liang Tao, Chunsheng Zhang, Zhiping Ying, Zhi Xiong, Haim Shalom Vaisman, Changming Wang, Zhuogong Shi, Rui Shi

**Affiliations:** ^1^Key Laboratory for Forest Resources Conservation and Utilization in the Southwest Mountains of China, Ministry of Education, Southwest Landscape Architecture Engineering Research Center of National Forestry and Grassland Administration, Southwest Forestry University, Kunming, Yunnan, China; ^2^Office of Academic Affairs, Yunnan University of Finance and Economics, Kunming, Yunnan, China; ^3^Yunnan Phosphate Haikou Co. LTD., Kunming, Yunnan, China

**Keywords:** *Macadamia integrifolia*, continuous monoculture, rhizosphere metagenomics, metabolomic profiling, physicochemical properties, bacterial diversity, metabolites

## Abstract

*Macadamia integrifolia* is the most economically important Proteaceae crop known for its edible nuts. The present study was conducted to examine the effect of continuous cultivation (for 1, 5, and 20 years) of *M. integrifolia* on soil quality, bacterial diversity, and metabolites. Soil rhizospheres from three different *Macadamia* rhizosphere orchards, 1-year monoculture orchard (CK), 5-year monoculture orchard (Y5), and 20-year monoculture orchard (Y20), were analyzed through metagenomic and metabolomic profiling. The soil physicochemical properties, including organic matter, and available nutrients (P, N, and K) were first increased significantly (*p* < 0.05) from the CK to the Y5 group and then decreased (*p* < 0.05) from the Y5 to the Y20 group. The soil pH continuously decreased (*p* < 0.05) over time from CK to Y20. Metagenomic profiling revealed that Actinobacteria, Acidobacteria, and Proteobacteria were the top three abundant phyla with their inconsistent relative abundance patterns from CK to Y20 (CK: 23.76%, Y5: 34. 06%, and Y20: 31.55%), (CK: 13.59%, Y5: 18.59%, and Y20: 21.35%), and (CK: 27.59%, Y5: 15.98%, and Y20: 17.08%), respectively. Furthermore, the Y5 rhizospheres had a higher number of beneficial bacterial genera belonging to Proteobacteria and Actinobacteria than the Y20 rhizospheres. The KEGG annotation analysis revealed that cellular processes, organism systems, metabolism, and genetic information were the most enriched functional categories. CAZy database screening indicated the highest enrichment of glycoside hydrolases following the glycoside transferases and carbohydrate-binding modules. Differential metabolite analysis revealed the highest number of metabolites (11) in the Y5 group than in the Y20 group (6). It is concluded that continuous monoculture of *M. integrifolia* improves the soil physicochemical properties, bacterial diversity, and metabolite contents in short-term planted orchards which, however, are deteriorated in long-term planted orchards.

## Introduction

Soil microbial communities are key determinants of soil health and can be altered by agricultural practices, including crop monoculture, rotation, soil tillage, irrigation, fertilization, and pesticide application (Tahat et al., 2020). Under intensive orchard management, continuous cropping is very common, which leads to changes in the soil nutrient levels, organic matter content, and, subsequently, the microbial communities. Soil quality and plant development can be affected positively or negatively by the composition and diversity of soil microorganisms (Köberl et al., 2013). The rhizosphere may contain about 30,000 microbial species and can harbor up to 1,011 microbial cells per gram of root (Berendsen et al., 2012). Root exudates attract microbes, and their composition is determined by exudate contents (Compant et al., 2010). The microbiome of each plant depends on its soil structure (Smalla et al., 2001).

Characterization of rhizosphere microbial communities can be performed using metagenomic sequencing technology (Zhou et al., 2021). The 16s rDNA gene sequencing technology has been applied for the exploration of microbial diversity in different environments (Youssef et al., 2009; Caporaso et al., 2011; Shi et al., 2021). Moreover, metabolomic profiling can be used to determine the important microbial metabolites affecting plant growth and development, and soil physicochemical properties (Grim et al., 2019). Rhizosphere metabolomics is an advanced technique that includes an unprejudiced examination of the complete metabolite profile (metabolome) for a better understanding of complex physiological, pathological, symbiotic, and other interactions between the microbial communities present in the rhizosphere soil (Reuben et al., 2008).

The genus *Macadamia* comprises four species of trees, including *Macadamia integrifolia*, *Macadamia tetraphylla*, *Macadamia jansenii*, and *Macadamia ternifolia*, and is native to Australia (Mast et al., 2008). It belongs to the plant family Proteaceae and is known for its edible nuts (Peace et al., 2003; Wallace and Walton, 2011). *Macadamia* nuts were first introduced to China in the 1970s from Australia. By the end of 2018, China’s *Macadamia* cultivated area surpassed 3,012.06 km^2^, accounting for more than one-third of the world’s planted area (Ma et al., 2020). China has become the world’s largest and fastest-growing *Macadamia* plantation country. In China, *Macadamia* nut production has exceeded thousands of tons with an average production of 1–5 tons per hectare in most orchards. The *M. integrifolia* plant is mainly grown in southern China, including the Yunnan and Guangxi provinces (Hong et al., 2018; Zhong et al., 2020). It is consumed as a snack and is also added to candies, cakes, and cookies. It is considered as one of the most essential nuts in the world, owing to its great nutritional content. *M. integrifolia* seeds contain 66.16 g of lipids, 8.40 g of proteins, and 1.36 g of sugars per 100 g of edible proportion (Venkatachalam and Sathe, 2006). Moreover, *Macadamia* nuts contain a substantial amount (77 g per 100 g of edible proportion) of monounsaturated fatty acids (MUFA), which can decrease the incidence of cardiovascular diseases in humans by decreasing the blood cholesterol levels, if consumed with a balanced diet (Garg et al., 2003; Griel et al., 2008). A new protein was found in *Macadamia* nuts called MiAMP1 protein, which imparts a robust ability to resist diseases (McManus et al., 1999). In transgenic canola, MiAMP1 gene expression showed great resistance against a fungal disease called blackleg, caused by *Leptosphaeria maculans* (Kazan et al., 2002).

Soil microorganisms belonging to distinct phylogenetic lineage vary in their response to changes in environmental conditions, thus changes in the soil nutrient profile during long-term monoculture cropping can influence the composition of the soil microbiome (Yan et al., 2021). Continuous monoculture can cause perturbation to the initial soil microbiome, leading to a positive and/or negative impact on plant health (Xiong et al., 2015; Fu et al., 2017; Zhao et al., 2020). Importantly, long-term monoculture rhizospheres exhibited distinct microbial communities than short-term monoculture soils in an orchard. Furthermore, continuous cropping in orchards may significantly alter the soil organic matter content, leading to a change in soil microbial communities. However, the impact of monoculture cropping of *M. integrifolia* on the rhizosphere microbiome and metabolites at different time points has never been elucidated so far, especially in a forest ecosystem.

Since continuous cropping affects soil microclimatic conditions, the present study aimed to investigate the impact of short-term (5 years) and long-term (20 years) continuous cropping of *M. integrifolia* orchards on the nutrient content, soil microbiome, and metabolites. The soil pH, nutrient content, and organic matter were analyzed in 1-year, 5-year, and 20-year orchards along with the changes in microbial community diversity and composition. We hypothesized that (1) the continuous monoculture of *M. integrifolia* plants will increase the soil available nutrients (N, P, and K) and organic matter due to the accumulation of litter from vegetation on the orchard floor over the years, (2) continuous monoculture with different years would have a differential effect on soil microbial community richness and composition, and (3) the soil metabolites in continuous monoculture orchards would be distinct from those of 1-year old orchards. To the best of our knowledge, this is the first study to examine soil microbiome using shotgun metagenomics and determine its association with soil metabolites under short-term and long-term continuous monoculture orchards of *M. integrifolia*.

## Materials and methods

### Experimental site and collection of rhizosphere soil samples

The experimental site was located in the Macadamia variety comparison test base of the Yunnan Institute of Tropical Crops, Yunnan Province, China (100°75′ E, 22°00′ N). This region has a subtropical climate with an annual precipitation of 950–1,000 mm and an average annual temperature of 22.6°C, while the annual evaporation is 1,310.6 mm. The experimental field soil at the test site was brick red loam soil. The experiment included three different treatments of *M. integrifolia* continuous monoculture orchards: (1) control (CK), a 1-year Macadamia tree grown field soil; (2) short-term monoculture (Y5), a 5-year Macadamia tree grown field soil; and (3) long-term monoculture (Y20), a 20-year Macadamia tree grown field soil to evaluate their impact on soil physicochemical properties, rhizosphere microbiome, and metabolites. For the rhizosphere microbiome analysis, a completely randomized design (CRD) was applied with three treatments, and each treatment was replicated three times, so a total of nine samples (three samples from each treatment) were collected randomly (*CK* = *CK.1*, *CK.2*, and *CK.3*; *Y5* = *Y5.1*, *Y5.2*, and *Y5.3*; and *Y20* = *Y20.1*, *Y20.2*, and *Y20.3*). For the collection of rhizosphere soil samples, a 5-point (W) sampling method was used. For each treatment, a total of 15 trees were selected randomly, and each replicate consisted of a mixture of rhizosphere soils from five trees. The loosely attached soil around the root area was shaken and collected into the zip lock bags (Smalla et al., 1993). All samples were put into the dry ice box and brought to the lab. Each soil sample was passed through a 2-mm sieve and then divided into two subsamples: one portion was used for the determination of soil physicochemical properties, while the remaining portion was kept at −80°C for microbiome analysis later.

### Determination of soil physicochemical properties

The analysis of the physicochemical properties of the rhizosphere soil was performed, as reported previously (Zheng, 2013). The pH of rhizosphere soil was determined by using a pH meter (FE-20, Swiss Mettler). The dichromate chemical oxygen demand test was applied to explore the organic matter (OM) content of the soil. Methods like the ammonium acetate extraction the flame photometry method, the diffusion method, and the Olsen method were performed for the quantification of available potassium (AK), nitrogen (AN), and phosphorus (AP), respectively.

### Determination of fruit yield of *Macadamia integrifolia*

By the end of August and the beginning of September, after the fruit was mature, the 5-point sampling method was used to collect the fruit in the production area. Briefly, one point was selected in each of the four corners and the center area, while side trees were not allowed to be selected, and six single plant samples were selected from each point. All the fruits were picked from each tree and weighed separately, and then, the average yield per plant and per unit area was calculated (kg per 666.7 m^2^ area).

### Rhizosphere sample collection and extraction of DNA

The roots of *M. integrifolia* were gently shaken to remove loosely attached soil particles and then vortexed in 50 ml tubes containing 0.1% Tween 20 solution for 5 min to wash away the firmly attached rhizosphere soil and assist soil descent. After carefully removing the roots, the soil suspension in the tube was centrifuged at 12,000 × g for 15 min. The rhizosphere soil was recovered after discarding the supernatant. The Power Soil DNA Kit (MOBIO Inc., Carlsbad, CA, USA) was used to extract the DNA from soil samples (0.5 g) by following the manufacturer’s instructions. For the quantification of DNA, a Qubit kit (Invitrogen, USA) was employed.

### DNA library construction and metagenomic sequencing

For the DNA library construction, a total of 1 μg of DNA per sample was utilized as input material. Following the manufacturer’s guidelines, sequencing libraries were created using the NEBNext Ultra DNA Library Prep Kit for Illumina (NEB, USA), and each sample was assigned sequences by adding index codes. Before PCR amplification and Illumina sequencing, the DNA material was first sonicated to 350-bp fragment size, then end-polished, A-tailed, and finally ligated with full-length adaptors. Finally, the PCR products were purified, and libraries were sized using the AMPure XP system and Agilent2100 Bioanalyzer, respectively. Real-time PCR was used for the quantification of PCR products. Following the manufacturer’s instructions, the index-coded samples were clustered using a cBot Cluster Generation System. Illumina HiSeq PE150 platform was used for the sequencing of the constructed library, and paired-end reads were produced.

### Microbiome analysis

Using an Illumina HiSeq PE150 platform, a total of nine DNA samples were sequenced as 350-bp paired-end reads. The METAFILER pipeline was used to analyze the metagenomic reads. Metagenomic reads obtained using the shotgun sequencing technology were further quality filtered by deleting shorter reads (that were shorter than 70% of the original reads of 350-bp) had an observed accumulator error > 2 or an estimated accumulated error > 2.5 with a probability of 0.01 or had > 1 ambiguous position. The sdm software was used to trim the reads at the 3′ end having base quality below 20 in a 15-base window or if the cumulative error surpassed 2. A similarity search approach was employed in the MetaGeneMark for the estimation of the functional composition of each sample. Each sample’s genes and mixed assembly prediction were combined for deduplication, a gene catalog was created, and clean data of each sample were synthesized from the gene catalog to get the gene abundance information in each sample. The MicroNR library was used to create the species abundance table of different taxonomic levels using the gene catalog and species annotation data of each gene (Unigene). Furthermore, the metabolic pathways (KEGG) (Kanehisa et al., 2014), homologous gene cluster (eggNOG) (Huerta-Cepas et al., 2017), and carbohydrate enzyme (CAZy) (Cantarel et al., 2009) databases were explored for functional annotation by utilizing the gene catalog data. The beta diversity of microbial species was calculated using the unweighted pair-group technique with an arithmetic mean (UPGMA) clustering approach based on Bray–Curtis distance. Heat maps for microbial taxa and functional features were created using R software version 3.1.3 and color-coded by row Z-scores.

### Metabolite profiling and analysis

The rhizosphere soil samples were homogenized for 1.5 min at 30 Hz in a mixer mill (MM 400, Retsch, Hann, Germany) containing zirconium beads. A 100 mg sample from each replicate was weighted and extracted with 1.2 mL of aqueous methanol (70%). The extraction of each replicate (100 mg) was done overnight at 4°C. After centrifugation at 12,000 rpm for 10 min, the extracts were filtered and analyzed utilizing a UPLC-ESI-MS/MS system for ultra-performance liquid chromatography-tandem mass spectrometry (UPLC-MS/MS) analysis.

Annotation of identified metabolites was performed through the Kyoto Encyclopedia of Genes and Genomes (KEGG) database,^1^ and the annotated metabolites were then mapped to the KEGG Pathway database.^2^ The *p*-values of the hypergeometric test were used to examine the important pathways with significantly regulated metabolites, which were then introduced into metabolite set enrichment analysis. Variable significance in projection, VIP > 1, and absolute Log2 FC (fold change) > 1 were used to assess whether the metabolites were significantly regulated across the groups. VIP values were extracted using the R package MetaboAnalystR from the orthogonal partial least squares discriminant analysis (OPLS-DA) results. Before OPLS-DA, the data were log-transformed (log2) and mean-centered. A permutation test (200 permutations) was used to avoid overfitting. The “psych” package in R 3.4.0 was used to determine Spearman’s correlation index values among microbial taxa and metabolites. The *cor* function in R was used to construct the heat maps of Pearson correlation coefficients (PCCs) between samples by including the values of correlation coefficient > 0.7 and *p* < 0.05 only.

### Statistical analysis

The data regarding physicochemical properties, fruit yield, and the number of non-redundant genes were analyzed using the analysis of variance technique under a completely randomized design (CRD), and treatment means were compared using Tukey’s test.

## Results

### Soil physicochemical properties and nut yield of *Macadamia integrifolia*

The changes in the physicochemical properties of soil samples for 1-year monoculture orchard (CK), 5-year monoculture orchard (Y5), and 20-year monoculture orchard (Y20) are presented in Table 1. The soil pH decreased gradually (*P* < 0.05) with an increase in the cropping period from CK (5.40 ± 0.012) to Y20 (4.94 ± 0.032), whereas all other physicochemical parameters were increased (*p* < 0.05) from CK to Y5 but decreased (*p* < 0.05) from Y5 to Y20. Furthermore, a higher yield (*p* < 0.05) of *M. integrifolia* nuts (in kg) per plant, per unit area, and per hectare was observed for Y20 when compared to the Y5 group (Table 2). The 1-year monoculture orchard (CK) did not show fruit production.

**TABLE 1:** Physicochemical properties of rhizosphere soil of *M. integrifolia* treatment groups (CK = 1-year monoculture orchard, Y5 = 5-year monoculture orchard, and Y20 = 20-year monoculture orchard).

Basic physical and chemical properties of soil	Index (1 year) (CK)	Index (5 years) (Y5)	Index (20 years) (Y20)
Organic matter (g/kg)	10.30 ± 0.009^c^	29.17 ± 0.060^a^	17.46 ± 0.042^b^
Available nitrogen (alkaline hydrolyzed nitrogen) (mg/kg)	21.40 ± 0.003^c^	109.23 ± 0.760^a^	87.02 ± 0.850^b^
Available phosphorus (mg/kg)	3.60 ± 0.011^c^	36.64 ± 0.170^a^	22.16 ± 0.370^b^
Rapidly available potassium (mg/kg)	88.30 ± 0.007^c^	194.54 ± 0.330^a^	155.05 ± 0.190^b^
pH	5.40 ± 0.012^a^	5.31 ± 0.220^b^	4.94 ± 0.032^c^

Values in the same row with different superscripts differ significantly.

**TABLE 2:** Nut yield of *M. integrifolia* for all three monoculture cropping systems (CK = 1-year monoculture orchard, Y5 = 5-year monoculture orchard, and Y20 = 20-year monoculture orchard).

Treatment	Yield (kg) per plant	Yield (kg) per 666.7 m^2^	Yield (kg) per hectare
CK	0	0	0
Y5	5 ± 1^c^	100 ± 20^b^	1500 ± 250^a^
Y20	25 ± 5^c^	500 ± 75^b^	7500 ± 850^a^

Values in the same row with different superscripts differ significantly.

### Metagenomic sequencing, assembly, and annotation

**FIGURE 1:**
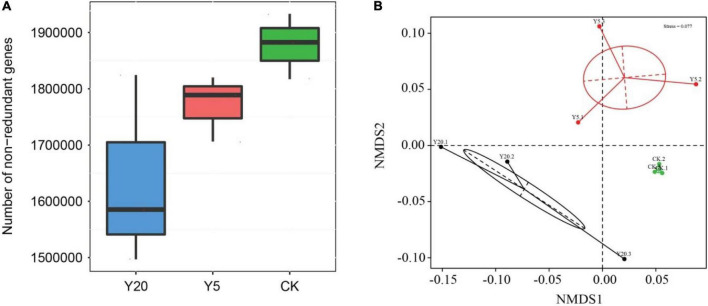
**(A)** A box plot showing non-redundant genes in *M. integrifolia* group samples (*CK* = 1-year monoculture orchard, *Y5* = 5-year monoculture orchard, and *Y20* = 20-year monoculture orchard). Boxes represent the interquartile ranges and lines represent the median values. **(B)** Non-metric multi-dimensional scaling (NDMS) analysis of all three treatments and their replicates (1-year monoculture orchard = *CK.1*, *CK.2*, and *CK.3*; 5-year monoculture orchard = *Y5.1*, *Y5.2*, and *Y5.3*; and 20-year monoculture orchard = *Y20.1*, *Y20.2*, and *Y20.3*). Each point represents different samples, and the distance between points indicates the degree of difference.

We assembled a total of 4,219,510 genes from nine shotgun metagenomes in the gene catalog. Among these, 1,406,102 (33.32%) genes contained only the start codon, 1,021,944 (24.22%) genes contained only the stop codon, 1,035,067 (24.53%) genes contained both start and stop codons, and 756,397 (17.93%) genes were without a start or terminating codon. The number of non-redundant bacterial genes was analyzed in all three groups (Figure 1A). The CK showed the highest number of non-redundant genes followed by Y5 and Y20 (Figure 1A). Furthermore, the highest variation in the number of non-redundant genes was observed for Y20 samples and the lowest for CK samples. The non-metric multi-dimensional scaling (NMDS) plot revealed that all three groups (CK, Y5, and Y20) were separated and that CK samples exhibited higher similarities between them, whereas Y5 and Y20 samples showed some obvious differences (Figure 1B).

### Bacterial diversity based on the eggNOG annotation

**FIGURE 2:**
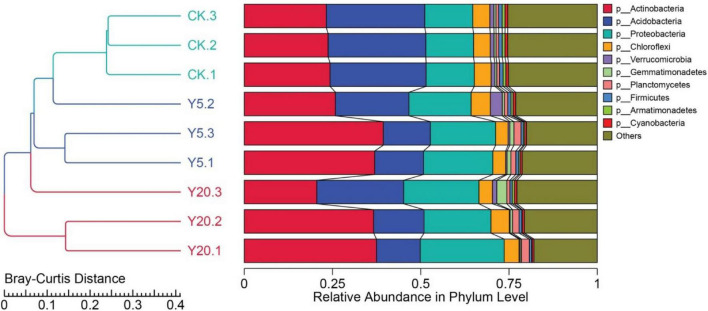
The phylogenetic relationship based on Bray–Curtis distance and histogram at the phylum level showing the composition and structure of the bacterial communities of *M. integrifolia* treatments [1-year monoculture orchard (CK) = *CK.1*, *CK.2*, and *CK.3*; 5-year monoculture orchard (Y5) = *Y5.1*, *Y5.2*, and *Y5.3*; and 20-year monoculture orchard (Y20) = *Y20.1*, *Y20.2*, and *Y20.3*].

We further annotated the genes from the metagenomes of *M. integrifolia* groups (CK, Y5, and Y20) to different taxonomic levels (from the phylum to species levels). The Bray–Curtis analysis clearly separated the samples into three groups: CK, Y5, and Y20 (Figure 2). We noted clear differences in CK vs. Y5 and CK vs. Y20, whereas higher similarities in Y5 vs. Y20 were observed. At the phylum level, Actinobacteria, Acidobacteria, and Proteobacteria were the top three dominant phyla in all three rhizosphere groups (CK, Y5, and Y20), as seen in Figure 2. The relative abundance of Actinobacteria (CK: 23.76%, Y5: 34.06%, and Y20: 31.55%) first increased from CK to Y5 and then decreased from Y5 to Y20. However, the relative abundance of Proteobacteria (CK: 13.59%, Y5: 18.59%, and Y20: 21.35%) was increased from CK to Y20. In contrast, the relative abundance of Acidobacteria (CK: 27.59%, Y5: 15.98%, and Y20: 17.08%) was first decreased from CK to Y5 and then increased from Y5 to Y20.

**FIGURE 3:**
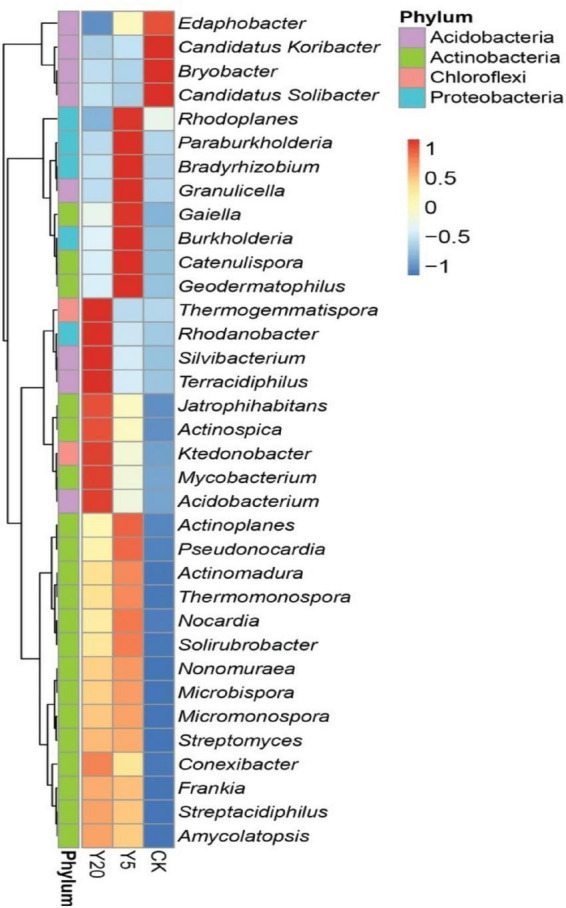
A heat map showing the top 35 most abundant genera through using normalized abundance in each *M. integrifolia* rhizosphere treatment group (*CK* = 1-year monoculture orchard, *Y5* = 5-year monoculture orchard, and *Y20* = 20-year monoculture orchard). The color codes are based on Z-scores.

Furthermore, the relative abundance of the top 35 genera for all three treatment groups (CK, Y5, and Y20) is presented in Figure 3. All three groups were differentially enriched with genera belonging to the top three phyla. Y5 had the highest (*p* < 0.05) enrichment than CK and Y20. Four genera, including Candidatus Koribacter, Edaphobactor, Bryobacter, and Candidatus Solibacter (phylum Acidobacteria), were highly abundant (*p* < 0.05) in the CK group. However, Rhodoplanes, Paraburkholderia, Bradyrhizobium, and Burkholderia (phylum Proteobacteria); Gaiella, Catenulispora, and Geodermatophilus (phylum Actinobacteria); and Granulicella (phylum Acidobacteria) were observed as dominant genera in the Y5 group. In the Y20 group, genera belonging to the phyla Acidobacteria (Silvibacterium, Terracidiphilus, and Acidobacterium), Actinobacteria (Jatriphihabitans, Actinospica, and Mycobacterium), and Proteobacteria (Rhodanobacter) were found to be highly (*p* < 0.05) enriched.

### Functional annotation using KEGG, eggNOG, and CAZy databases

**FIGURE 4:**
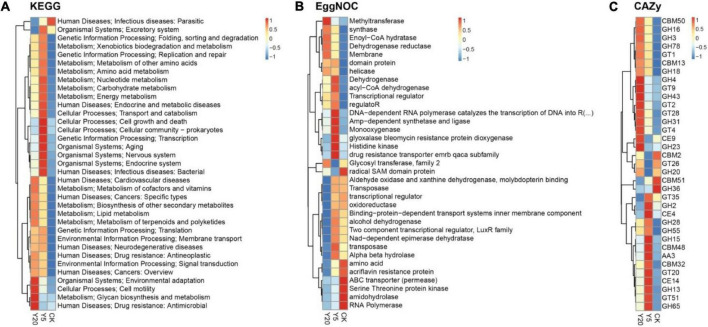
A cluster heat map showing the abundance of the differential functions. The results of KEGG **(A)**, eggNOG **(B)**, and CAZy **(C)** are displayed from top to bottom: landscape for the treatment group, vertical right information for functional annotation, vertical left information for functional clustering tree, and the Z-values (*CK* = 1-year monoculture orchard, *Y5* = 5-year monoculture orchard, and *Y20* = 20-year monoculture orchard).

The KEGG functional annotation analysis revealed genes highly enriched (*p* < 0.05) with functions related to infectious parasitic diseases in the CK group; cellular processes (cell growth and death, and prokaryotic cellular community), genetic information (processing: transcription), and organismal systems (aging, nervous system, and endocrine system) in the Y5 group; and cellular processes (cell motility), metabolism (glycan biosynthesis and metabolism), and antimicrobial drug resistance in the Y20 group (Figure 4A). Furthermore, the analysis of unigenes with known functions in the eggNOG database revealed a higher number of unigenes (*p* < 0.05) associated with enzyme functions like radical SAM domain protein, ABC transporters (permease), serine-threonine protein kinase, amidohydrolase, and RNA polymerase in the CK group; DNA-dependent RNA polymerase, AMP-dependent synthetase and ligase, monooxygenase, glyoxalase bleomycin protein dioxygenase, histidine kinase, and drug resistance transporter EmrB/QacA subfamily in the Y5 group and methyltransferase in the Y20 group (Figure 4B). Overall, in all three groups (CK, Y5, and Y20), the highest number of unigenes (*p* < 0.05) in the CAZy database was associated with glycoside hydrolases (GH) followed by the glycoside transferases (GT) and carbohydrate-binding modules (CBM), as seen in Figure 4C. Overall, the results showed the highest enrichment of gene functions in the Y5 group.

### Comparison of metabolite contents in different rhizosphere groups

**FIGURE 5:**
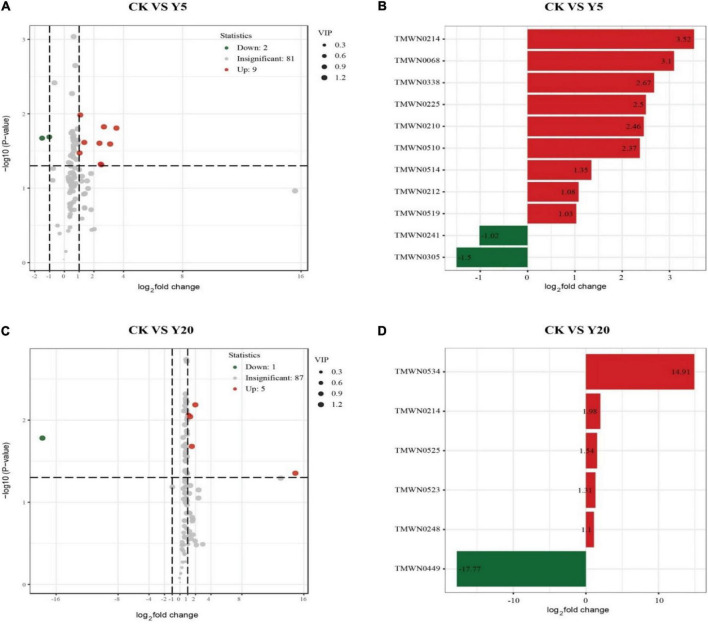
A volcano map **(A,C)**, the abscissa indicates the changes in the expression of multiple metabolites in different groups: **(A,B)** CK vs. Y5, **(C,D)** CK vs. Y20 (log2 fold change). The vertical coordinate indicates the differences in the significance level (–log10p). Each point in the volcano chart represents a metabolite, the significantly upregulated metabolite is represented by a red dot, and the significantly downregulated metabolite is represented by a green dot.

Metabolomic analysis revealed a total of 92 differential metabolites for CK vs. Y5, of which 11 (9 upregulated and 2 downregulated) were found to be significantly enriched (*p* < 0.05) (Figures 5A,B). Similarly, 93 differential metabolites were observed for CK vs. Y20, of which 6 (5 upregulated and 1 downregulated) were found to be significantly enriched (Figures 5C,D).

**FIGURE 6:**
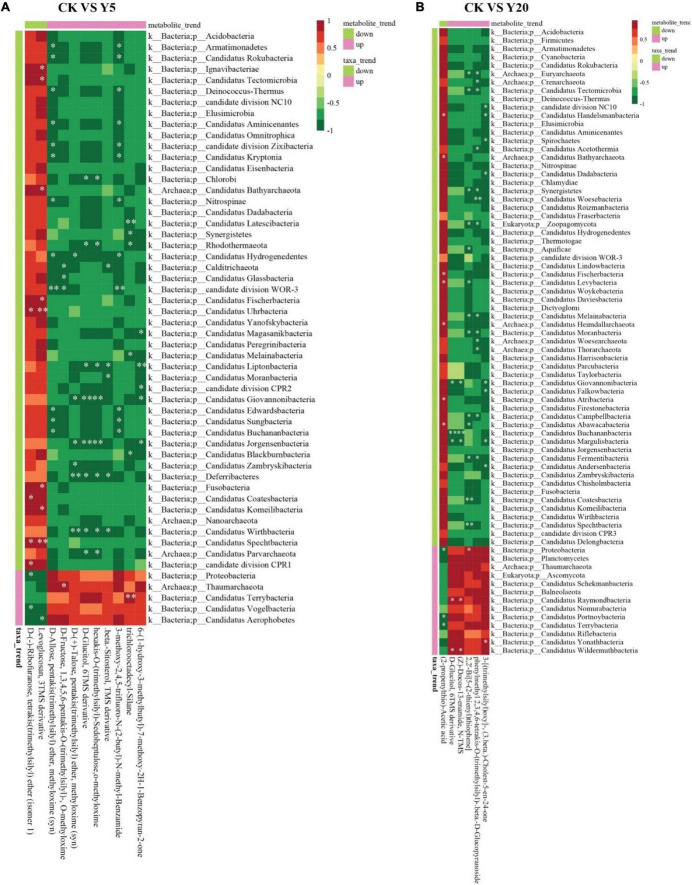
Spearman’s correlation between abundant bacterial genera and significant metabolites for CK vs. Y5 **(A)** and CK vs. Y20 **(B)**. The horizontal bottom is the observed metabolite information, the horizontal top is the metabolite trend (upregulated or downregulated), the vertical right is the bacterial genera information, the vertical left is the taxa trend information, and the corresponding value of the intermediate heat map is the Spearman correlation coefficient r (range = –1 to +1); r < 0 is a negative correlation, r > 0 is a positive correlation, and the * indicates the significance at a *p*-value < 0.05 and ** indicates the significance at a *p*-value < 0.01.

Furthermore, Spearman’s correlations were determined between the differential metabolites and bacterial phyla for CK vs. Y5 and CK vs. Y20 (Figures 6A,B). In the CK vs. Y5 group, a downregulated metabolite (levoglucosan, 3TMS derivative) showed a highly positive correlation (*p* < 0.01) with Candidatus Spechtbacteria and Candidatus Uhrbacteria (Figure 6A). Similarly, an upregulated metabolite (trichlorooctadecyl-silane) also showed a highly positive (*p* < 0.01) correlation with Candidatus Terrybacteria for CK vs. Y5. Moreover, a positive (*p* < 0.05) correlation was observed between a downregulated metabolite (2-propenylthio-acetic acid) and bacteria related to Candidatus phyla for CK vs. Y20 (Figure 6B). Similarly, upregulated metabolites [D-glucitol, 6TMS derivative, (Z)-docos-13-enamide, N-TMS, (2-propenylthio)-acetic acid, and phenylmethyl 2,3,4,6-tetrakis-*O*-(trimethylsilyl)-beta-D-glucopyranoside] exhibited positive (*p* < 0.05) correlation with bacteria related to Candidatus phyla for CK vs. Y20.

## Discussion

### Soil physicochemical properties

Long-term monoculture cropping is a common practice in agriculture, but it may cause problems like a decrease in crop yield (Vargas Gil et al., 2009) and soil quality (Wu et al., 2015) and can also lead to plant diseases (Shipton, 1977). Previous studies demonstrated that continuous monoculture might increase the soil’s physicochemical properties initially but then deteriorate with a continuous cropping period (Xiong et al., 2015; Fu et al., 2017; Zhao et al., 2020). The present study also revealed significant improvement in soil physicochemical properties of *M. integrifolia*, including pH, OM, AN, AP, and AK, from CK to Y5, whereas these parameters decreased from Y5 to Y20. Previous studies also demonstrated the same trend in physicochemical properties under continuous mono-cropping, and a positive correlation was detected between physicochemical properties and microbial diversity (Xiong et al., 2015; Fu et al., 2017; Zhao et al., 2020). Unlikely, we observed an increase in nut yield (kg) in Y20 compared to CK and Y5. The major reason behind this higher nut yield of *Macadamia* in Y20 irrespective of the decrease in soil physicochemical parameters is attributed to the fact that the *Macadamia* plant starts nut production at around 4 years of age and attains maturity at 12 or 14 years of age, so at 20 years of age, it will have higher production than Y5 (when it starts fruiting). As our findings indicated deterioration in soil quality in long-term *Macadamia* continuous monoculture, it might continue to degrade further and also affect yield in the later years. Therefore, soil management for *M. integrifolia* cropping is essentially required to maintain the soil quality, maintain microbial diversity, and sustain nut production. One of the ways to improve these parameters is by adopting intercropping management system. Previously, *Macadamia* intercropping with coffee yielded good economic returns while improving soil quality and biodiversification (Perdoná and Soratto, 2015, 2016, 2020). In southern China, farmers have started *Macadamia* intercropping with coffee, maize, banana, and tea. *Macadamia* intercropping with maize decreased the soil temperature and improved fertilizer use and nutrient availability in hot summers (Zhao X. et al., 2019).

### Metagenomic profiling of bacterial communities

The metagenomic profiling of rhizospheres of *M. integrifolia* (CK, Y5, and Y20) revealed that the highest number of non-redundant genes was present in CK and the lowest in Y20. Further, the NDMS analysis revealed that all three treatment groups had significant differences. The present study revealed the same top 10 bacterial phyla across the three groups (CK, Y5, and Y20), but their relative abundance varied between them. The Y5 and Y20 groups had more similar bacterial communities than those observed in the CK group. Actinobacteria, Acidobacteria, and Proteobacteria were the top three abundant bacterial phyla across the three treatment groups. These top three bacterial phyla were also found abundantly in the previous studies conducted in Yunnan province (Tan et al., 2017; Yang et al., 2020, 2021; Kui et al., 2021; Zhu et al., 2021); however, Proteobacteria was the most dominant bacterial phyla in these studies. The relative abundance of Actinobacteria was first increased from CK to Y5 and then decreased from Y5 to Y20. Actinobacteria are involved in carbon cycling and decomposition of soils (Fradin and Thomma, 2006; Upchurch et al., 2008). They also play an important role in disease suppression (Priyadharsini and Dhanasekaran, 2015), as disruption of these bacteria resulted in wilt disease in tomatoes (Lee et al., 2021). Furthermore, Actinobacteria can survive under severe distress (Simmons et al., 2020), so an increase in the abundance of Actinobacteria might have played role in improving soil physicochemical properties, owing to its crucial role in carbon cycling, soil decomposition, and release of important nutrients. The relative abundance of Proteobacteria was increased from CK to Y20 in the present study. Proteobacteria are associated with the recycling of different nutrients like N, S, P, Fe, and C in rhizosphere soils (Hedrich et al., 2011; Li et al., 2014), and an increased abundance of Proteobacteria in rhizosphere soils is linked with higher nutrient levels (Smit et al., 2001; Liu et al., 2018). The relative abundance of Acidobacteria was first decreased from CK to Y5 and later increased from Y5 to Y20. Acidobacteria are found mostly in soils with low pH and nutrient levels (Barns et al., 1999) and are involved in the degradation of C utilizing cellulose, hemicellulose, and chitin (De Chaves et al., 2019). In the present study, the decrease in the relative abundance of Acidobacteria in Y5 regardless of the decrease in pH might be attributed to the change in the other physicochemical parameters, which further decreased in Y20. Overall, the increased enrichment of beneficial bacterial communities in Y5 than that observed in CK and Y20 might be associated with an improvement in the physicochemical properties of rhizosphere soils from CK to Y5, owing to the positive correlation between microbial diversity and physicochemical parameters (Xiong et al., 2015; Fu et al., 2017; Zhao et al., 2020).

The results of bacterial diversity at the genus level revealed that Y5 was the highly diverse group containing significant bacterial genera related to the phyla Actinobacteria and Proteobacteria. In the Y5 group, genera belonging to the phyla Proteobacteria (Rhodoplanes, Paraburkholderia, and Bradyrhizobium) and Actinobacteria (Gaiella, Catenulispora, and Geodermatophilus) were found to be highly (*p* < 0.05) enriched. The genus Rhodoplanes has been detected in *Panax notoginseng* rhizosphere soil continuous monoculture (Tan et al., 2017) and is well-known as a potential N fixer (Buckley et al., 2007). The relative abundance of the genus Paraburkholderia was increased in response to the continuous monoculture cropping of cucumber (Li et al., 2021), and its role in sustainable crop production and environmental protection is well-established (Kaur et al., 2017). The relative abundance of Bradyrhizobium was decreased in the long-term continuous monoculture of *Pinellia ternate*, and it was suggested that it might be important for soil fertility and the removal of phenolic compounds (Zhao et al., 2021). The genus Gaiella had shown a significant positive correlation with the continuous monoculture of *Medicago sativa L*. (alfalfa), whereas a significant negative correlation was observed with total phosphorus and available phosphorus content (Xu et al., 2022). Beneficial taxa belonging to the genus Gaiella have been shown to suppress Fusarium wilt (Zhao F. et al., 2019). The genus Geodermatophilus plays an important role against abiotic stresses (Montero-Calasanz et al., 2012, 2013), and its abundance was decreased in sugarcane monoculture (Tayyab et al., 2021). In the Y20 group, the abundance of Silvibacterium, Terracidiphilus, and Acidobacterium (phylum Acidobacteria) and of Rhodanobacter (Proteobacteria) was increased. Silvibacterium genus belongs to Acidobacteria subdivison 1 (sd1), and its abundance was increased in the root rot rhizosphere soil of *Persea americana Mill.* (avocado) (Shu et al., 2019). Terracidiphilus genus also belongs to sd1 and can survive at low pH values. It has been reported to play an important role in organic matter transformation (García-Fraile et al., 2016). In contrast to our study, Rhodanobacter genus increased in 10-year continuous monoculture of tea, whereas it decreased in 20-year monoculture tea orchard and exhibited a negative correlation with pH and positive correlation with N content and AK (Li et al., 2016). From these findings, it can be inferred that beneficial bacterial communities increased in short-term monocultures (Y5), and later, they started to deteriorate in long-term monocultures (Y20), which had more acidophilic bacteria. Furthermore, the increase in the abundance of Acidobacteria in Y20 might be attributed to the decrease in soil pH values.

### Functional annotation using KEGG, eggNOG, and CAZy databases

In the present study, Y5 rhizospheres exhibited the most diversely enriched functions and enzyme pathways than CK and Y20 as revealed through KEGG eggNOG and CAZy analyses. Overall, major pathways were related to cellular processes, genetic information, organismal systems, and metabolism in CK, Y5, and Y20. Furthermore, the analysis of unigenes with known functions in the eggNOG database revealed a higher number of unigenes (*p* < 0.05) associated with enzymes like radical SAM domain protein, ABC transporter (permease), serine-threonine protein kinase, amidohydrolase, and RNA polymerase in the CK group. However, DNA-dependent RNA polymerase, AMP-dependent synthetase and ligase, monooxygenase, glyoxalase bleomycin protein dioxygenase, histidine kinase, and drug resistance transporter EmrB/QacA subfamily were the main pathways in the Y5 group and methyltransferase in the Y20 group. Collectively, CAZy database screening revealed the highest number of unigenes linked with glycoside hydrolases (GH) following glycoside transferases (GTs) and carbohydrate-binding module (CBM). Glycoside hydrolases catalyze the hydrolysis of glycosidic bonds in complex sugars like cellulose and hemicellulose (Henrissat and Davies, 1997; Bourne and Henrissat, 2001). They are also involved in antibacterial defense strategies, pathogenesis, and normal cellular function. The CAZy database contains more than 120 families of GH (Cantarel et al., 2009). Glycoside transferases play an important role in the biosynthesis of disaccharides, oligosaccharides, and polysaccharides by catalyzing the transfer of sugar moieties from activated donor molecules to other sugar molecules (Schmid et al., 2016). GTs have more than 90 families in the CAZy database (Cantarel et al., 2009). Carbohydrate-binding module is a protein present in GT and GH and carbohydrate-binding activity. By binding to certain plant structural polysaccharides, CBMs of microbial GH play an important role in the recycling of photosynthetically fixed carbon (Szabó et al., 2001). CBM has more than 60 families present in the CAZy database (Cantarel et al., 2009). The GH and GT have been shown to be important in the metabolic activity of *M. integrifolia* microbial populations, so *M. integrifolia* might adapt effectively under harsh conditions with the use of diverse metabolic potential.

### Differential metabolite analysis

Differential metabolite analysis revealed that the CK vs. Y5 group had 11 significant metabolites, whereas the CK vs. Y20 group had 6 significant metabolites. Therefore, a significant number of metabolites were decreased in Y20. Furthermore, Spearman’s correlation at the phylum level revealed that levoglucosan, a 3TMS derivative (downregulated metabolite), had a highly significant positive correlation with Candidatus Spechtbacteria and Candidatus Uhrbacteria for CK vs. Y5. Levoglucosan is a combustion product of cellulose and produced only when it is heated over 300°C and is used as a tracer compound for biomass burning (Bae et al., 2012). The presence of levoglucosan in the rhizosphere soil of *M. integrifolia* might indicate the *in situ* burning of agricultural waste in the past. Similarly, trichloro-octadecyl-silane (upregulated metabolite) also showed a highly significant correlation with Candidatus Terrybacteria for CK vs. Y5. Moreover, a significant positive correlation was observed between a downregulated metabolite called (2-propenylthio)-acetic acid and bacteria related to Candidatus phylum for the CK vs. Y20 group. Similarly, upregulated metabolites [D-glucitol, 6TMS derivative, (Z)-docos-13-enamide, N-TMS, (2-propenylthio)-acetic acid, and phenylmethyl 2,3,4,6-tetrakis-*O*-(trimethylsilyl)-beta-D-glucopyranoside] had significant correlation with bacteria related to Candidatus phylum for CK vs. Y20 group. Glucitol or sorbitol is a 6-carbon sugar compound found in many fruits, and bacteria can utilize glucitol as a carbon source through the catabolic pathway during glycolysis (Li et al., 2022). (Z)-Docos-13-enamide, also known as erucamide, is a fatty acid amide that has an important role in nitrogen metabolism in the rhizosphere bacteria (Sun et al., 2016). Erucamide is involved in nitrogen removal by utilizing two denitrifying bacteria containing nitrate and nitrite reductases. Acetic acid is a weak carboxylic acid and possesses phytotoxic properties when released after the anaerobic decomposition of organic matter (Lynch, 1977). Glucopyranoside has been observed in petroleum-contaminated soil with a potent ability to reduce total petroleum hydrocarbons (TPHs) (Wang et al., 2016). Our findings suggest using the intercropping system for *M. integrifolia* cropping to maintain the soil quality and bacterial diversity, as demonstrated previously (Zhao X. et al., 2019). Currently, China is the leading *Macadamia* planting country; thus, management strategies for sustainable *Macadamia* orchards are urgently required.

## Conclusion

The present study revealed that the continuous monoculture of *M. integrifolia* improved soil physicochemical properties, including OM, AP, AN, and AK, in short-term monoculture orchards but deteriorated in long-term monoculture orchards. Rhizosphere soil metagenomic profiling revealed that Actinobacteria, Acidobacteria, and Proteobacteria were the top three abundant phyla. Metabolomic profiling revealed differential enrichment of metabolites in short-term and long-term monoculture orchards. Our study concluded that the continuous monoculture of *M. integrifolia* improved the soil physicochemical properties, enrichment of more beneficial bacterial communities, and metabolites for short-term monoculture cropping, which later on decreased for long-term monoculture cropping. These findings indicated that rhizosphere soil management is important for sustainable crop production and efficiency. It is suggested that the intercropping system for *M. integrifolia* should be adopted to maintain the soil quality and bacterial diversity for sustainable nut production.

## Data availability statement

The data presented in the study are deposited in the NCBI SRA repository, accession number PRJNA883021 (https://www.ncbi.nlm.nih.gov/bioproject/?term=PRJNA883021).

## Author contributions

LT and CZ: conceptualization. ZY and ZX: data curation. ZY, ZX, and CW: formal analysis. CW, ZS, and RS: funding acquisition. HV and ZY: investigation. CZ, ZY, ZX, and ZS: methodology. CZ and ZX: project administration. LT: resources. LT, CZ, and CW: software. ZS and HV: validation. ZS and CZ: visualization. CW and ZS: writing of the original draft. RS: reviewing and editing. All authors have read and agreed to the published version of the manuscript.

## Footnotes

1 http://www.kegg.jp/kegg/compound/, accessed on 10 August 2021.

2 http://www.kegg.jp/kegg/pathway.html, accessed on 10 August 2021.
